# Mutualism Breakdown by Amplification of *Wolbachia* Genes

**DOI:** 10.1371/journal.pbio.1002065

**Published:** 2015-02-10

**Authors:** Ewa Chrostek, Luis Teixeira

**Affiliations:** Instituto Gulbenkian de Ciência, Oeiras, Portugal; Fred Hutchinson Cancer Research Center, UNITED STATES

## Abstract

Most insect species are associated with vertically transmitted endosymbionts. Because of the mode of transmission, the fitness of these symbionts is dependent on the fitness of the hosts. Therefore, these endosymbionts need to control their proliferation in order to minimize their cost for the host. The genetic bases and mechanisms of this regulation remain largely undetermined. The maternally inherited bacteria of the genus *Wolbachia* are the most common endosymbionts of insects, providing some of them with fitness benefits. In *Drosophila melanogaster*, *Wolbachia* *w*MelPop is a unique virulent variant that proliferates massively in the hosts and shortens their lifespan. The genetic bases of *w*MelPop virulence are unknown, and their identification would allow a better understanding of how *Wolbachia* levels are regulated. Here we show that amplification of a region containing eight *Wolbachia* genes, called Octomom, is responsible for *w*MelPop virulence. Using *Drosophila* lines selected for carrying *Wolbachia* with different Octomom copy numbers, we demonstrate that the number of Octomom copies determines *Wolbachia* titers and the strength of the lethal phenotype. Octomom amplification is unstable, and reversion of copy number to one reverts all the phenotypes. Our results provide a link between genotype and phenotype in *Wolbachia* and identify a genomic region regulating *Wolbachia* proliferation. We also prove that these bacteria can evolve rapidly. Rapid evolution by changes in gene copy number may be common in endosymbionts with a high number of mobile elements and other repeated regions. Understanding *w*MelPop pathogenicity and variability also allows researchers to better control and predict the outcome of releasing mosquitoes transinfected with this variant to block human vector-borne diseases. Our results show that transition from a mutualist to a pathogen may occur because of a single genomic change in the endosymbiont. This implies that there must be constant selection on endosymbionts to control their densities.

## Abstract 2

An elegant experimental evolution approach reveals that a strain of the symbiotic bacterium Wolbachia that over-replicates and shortens the life of its fruit fly host owes this property to the amplification of a small region of its genome. Read the accompanying Primer.

## Author Summary

Insects frequently carry intracellular bacteria that are passed from generation to generation through their eggs. These intracellular symbionts can be beneficial or parasitic, but because of their mode of transmission, they are always dependent on the reproduction of their carriers. They therefore have to control their own growth in order to minimize deleterious effects on the host. Bacteria of the genus *Wolbachia* are the most common maternally transmitted intracellular bacteria in insects. Most *Wolbachia* variants that are naturally associated with the fruit fly *Drosophila melanogaster* are benign to their hosts and provide them with protection against viruses. However, *w*MelPop is a virulent *Wolbachia* variant that over-replicates massively and shortens the lifespan of its fruit fly host. Here we show that amplification of a *Wolbachia* genomic region containing eight genes—called Octomom—is responsible for the pathogenic effects of *w*MelPop. Our results provide a link between genotype and phenotype in *Wolbachia* and show that virulence in symbionts can be simply caused by increases in gene copy number. These results also indicate that gene copy number variation may be a common mechanism underlying rapid evolution of intracellular symbionts.

## Introduction

Vertically transmitted bacterial endosymbionts are widespread in arthropods, particularly in insects [1]. Many endosymbionts are mutualists and confer a fitness advantage to the host. The benefits may range from metabolic provisioning to protection against pathogens [2]. Other symbionts act as parasites and manipulate host reproductive biology in order to increase the relative fitness of their carriers [3]. In both cases, the density of endosymbionts within hosts is a crucial factor determining their prevalence in host populations [4,5].

Symbiont densities are determined by host and symbiont genetic diversity and environment [4,6–10]. These densities are under selection at the level of the host and the symbiont. Interestingly, there are conflicting selective forces at the level of the symbiont. Higher symbiont densities are associated with higher transmission fidelity and stronger phenotypes induced in the host [4,5,8,11–17]. Theoretically, this should lead to a selection for higher densities. On the other hand, high symbiont levels may have a negative impact on host fitness [8,9,17–19]. Since vertical transmission leads to dependence of the symbiont on the fitness of the host, it is advantageous for endosymbionts to limit their densities and consequently minimize the cost to their hosts. Thus, a key question in the field of host–microbe interactions is how symbionts regulate their replication and resulting densities to achieve an equilibrium between these opposing selective forces.

*Wolbachia* is conceivably the most prevalent bacterial endosymbiont of insects [20,21], and its interactions with hosts have been studied extensively. *Wolbachia* is maternally transmitted and exhibits a range of phenotypes. These include cytoplasmic incompatibility and other reproductive manipulations that potentiate *Wolbachia* spread in host populations [22]. Some *Wolbachia* strains have also been shown to be metabolic mutualists [23] or to protect insects from viral infections [24–27]. The *Wolbachia* strain infecting *Drosophila melanogaster*, *w*Mel, exerts only a weak cytoplasmic incompatibility in laboratory conditions [28], and this reproductive manipulation seems not to be expressed in field conditions [29]. Since cytoplasmic incompatibility cannot explain *Wolbachia* prevalence in *D*. *melanogaster* populations [28,30], it was suggested that *w*Mel exerts positive fitness effects on its hosts [29]. More recently, it was shown that *w*Mel provides *Drosophila* with strong resistance to systemic and oral infection with the natural pathogen *Drosophila* C virus (DCV) [24,25,31]. This protection extends to RNA viruses of different families [24,25,27,32], indicating that *w*Mel protects against a wide range of RNA viruses. Some of the fastest evolving genes in *D*. *melanogaster* are involved in antiviral RNA interference and are under strong positive selection [33]. Therefore, viruses seem to be a strong selective force in *D*. *melanogaster*. Moreover, several viruses, including DCV, have been isolated from natural populations of *D*. *melanogaster* [34–36]. Although there are no data regarding *Wolbachia* antiviral protection in natural populations, the *D. melanogaster–w*Mel–DCV interaction fulfills many of the criteria for defensive mutualism [37]. Therefore, antiviral protection may be the cause of *w*Mel maintenance in *D*. *melanogaster* natural populations.

Natural *w*Mel variants have a small effect on host longevity, yet they provide a strong antiviral protection [8]. This protection is positively correlated with *Wolbachia* density: the higher the titers of *Wolbachia*, the higher the antiviral protection [8,17,18,26,38–40]. On the other hand, high endosymbiont densities can have a cost in the absence of viral infection, and *Wolbachia* variants conferring strong protection often shorten the lifespan of the flies [8,18]. There is thus a fine balance between density, benefit, and cost to the host.

The *w*Mel variant *w*MelPop breaks this balance and is clearly pathogenic: it over-proliferates and dramatically shortens the lifespan of infected flies [8,19,41,42]. *w*MelPop is, hence, an exceptional vertically transmitted symbiont. Its uniqueness was immediately recognized as providing a tool to better understand regulation of vertically transmitted symbionts and the biology of *Wolbachia* [43].

Understanding the cause of the *w*MelPop phenotype and regulation of *Wolbachia* densities is also important from an applied perspective. Several *Wolbachia* strains, including *w*MelPop, have been transinfected into mosquito vectors of human diseases, where they can interfere with arboviruses or other pathogens [44–52]. The purpose of this research is to release *Wolbachia*-carrying mosquitoes refractory to human pathogens into natural populations and prevent infections in humans. *Aedes aegypti* mosquitoes carrying *Wolbachia* are more resistant to dengue virus and are already being tested in the field [44,50,53–55]. Different variants of *w*Mel transinfected into *A*. *aegypti* show a trade-off between host fitness and resistance to dengue virus. A *w*MelPop-derived strain gives higher resistance to dengue but has a high fitness cost, which may prevent it from stably infecting natural mosquito populations [56,57]. On the other hand, a *w*Mel-derived strain confers lower protection to dengue virus but is able to stably invade *A*. *aegypti* populations [50,53–55]. Ideally, a further understanding of the system would allow researchers to use *Wolbachia* strains with a better ratio of antiviral protection to cost. Moreover, since *w*MelPop has been transinfected into mosquitoes [44–48], understanding the pathogenicity of this *Wolbachia* variant is crucial for predicting *w*MelPop dynamics in the released mosquito populations.

Finding the genetic basis of *w*MelPop pathogenicity is essential to understanding its phenotype. Difficulty in the functional analysis of *Wolbachia* lies in its refractoriness to genetic manipulation. Nonetheless, genomic analyses have provided insight into the cause of *w*MelPop pathogenicity. The first genomic map of *w*MelPop was published in 2003 [58], while the full genome of the similar *w*Mel was published in 2004 [59]. Analyses of polymorphic genomic markers and whole genome assemblies have shown that *w*MelPop is closely related to *w*MelCS variants [8,60–62]. We have recently identified genetic differences between *w*MelPop and the closely related non-pathogenic *w*MelCS_b [8]. The *w*MelPop genome contains an amplification of a ~21-kb region, named Octomom, that includes eight *Wolbachia* genes (*WD0507* to *WD0514*) flanked by direct repeats. This amplification in *w*MelPop was also described by Woolfit and colleagues [62]. Apart from this amplification, we found only one synonymous SNP unique to *w*MelPop (position 943,443, G>A) [8]. Therefore, we hypothesized that Octomom region amplification underlies *w*MelPop virulence. Gene amplification has previously been reported to change the pathogenicity of other bacteria and viruses [63–67].

Here we show strong evidence that, in support of our original hypothesis, Octomom region amplification is the cause of the *w*MelPop phenotypes of over-replication and pathogenicity.

## Results

**Fig 1:**
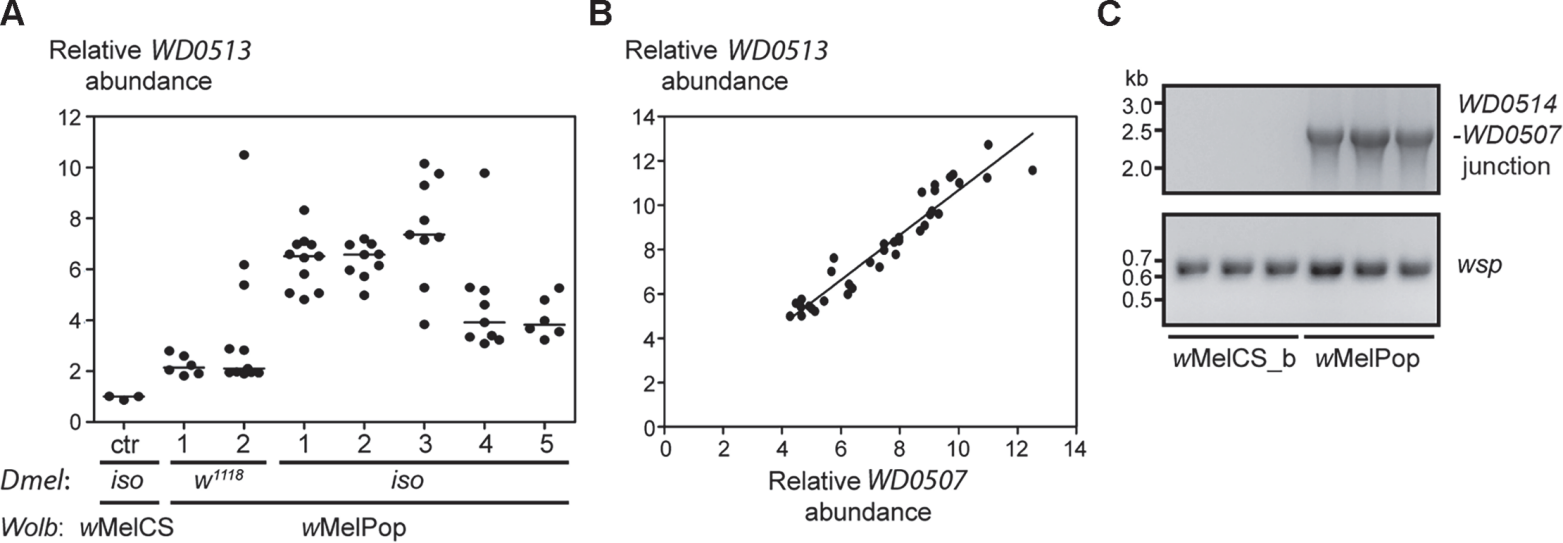
Individual *w*MelPop flies differ in Octomom copy numbers. (A) *WD0513* copy number variability in single females from two *w*MelPop stocks with *w* ^*1118*^ and *iso* genetic backgrounds, relative to *wsp*. We tested two replicates of *w* ^*1118*^ stock and five replicates of *iso* stock. *w*MelCS_b *iso* flies were used for copy number normalization (control [ctr]). Lines are medians of the replicates. Supporting data can be found in S1 Data. (B) Relation between *WD0507* and *WD0513* abundance in single *w*MelPop females. Each dot represents a female, and the regression line is shown. The estimates for the fitted regression line are slope = 1.036 ± 0.041, intercept = 0.182 ± 0.204, *R* ^2^ = 0.92. Supporting data can be found in S2 Data. (C) PCR of the predicted *WD0514–WD0507* junction in *w*MelPop flies. *w*MelCS_b was used as a negative control. Three samples of each *Wolbachia* variant were used. PCR for *wsp* gene was used as a DNA quality control.

Currently, *Wolbachia* cannot be genetically manipulated, which hinders functional studies on *Wolbachia* genes. However, bacterial amplified DNA sequences have been described before as unstable [64], leading us to test the hypothesis that natural variation in Octomom copy number exists and causes distinct phenotypes. To detect Octomom copy number variation, we tested several single *Drosophila* females for the copy number of the Octomom gene *WD0513* in their *Wolbachia* bacteria (Fig. 1A). The copy number of *WD0513* was determined by quantitative PCR on genomic DNA samples from single flies carrying *Wolbachia*, using *Wolbachia wsp* (*Wolbachia* surface protein) as a reference gene. *w*MelCS_b samples were used as reference samples for one *WD0513* copy, based on the coverage analysis of our previous *Wolbachia* sequencing data [8]. We analyzed two fly stocks infected with *w*MelPop: *w* ^*1118*^, derived from the original stock in the Benzer lab [19], and a DrosDel isogenic *w* ^*1118*^ (*iso*) stock into which we introgressed *w*MelPop from the *w* ^*1118*^ stock [8]. All *w*MelPop samples analyzed had at least a duplication of the Octomom region, with high variation in *WD0513* copy number between individual females, ranging from two to ten copies (Fig. 1A). This copy number corresponds to the average *WD0513* copy number in the *Wolbachia* of each individual female (thus, differences in Octomom copy number between *Wolbachia* cells within each female may exist).

To check whether the *Wolbachia* Octomom region is amplified as a unit, we tested *WD0507* and *WD0513* copy number simultaneously in individual flies. The copy numbers of the two genes are the same in each fly (Figs. 1B and S1), suggesting integrity of the Octomom region. A common mechanism of gene amplification in bacteria leads to tandem duplications and the formation of new junctions between units [64]. We detected the presence of this new predicted *WD0514–WD0507* junction by PCR and Sanger sequencing (Fig. 1C; S1 Text). These data show that Octomom copy number is highly variable and that the amplification is consistent with a tandem duplication.

**Fig 2:**
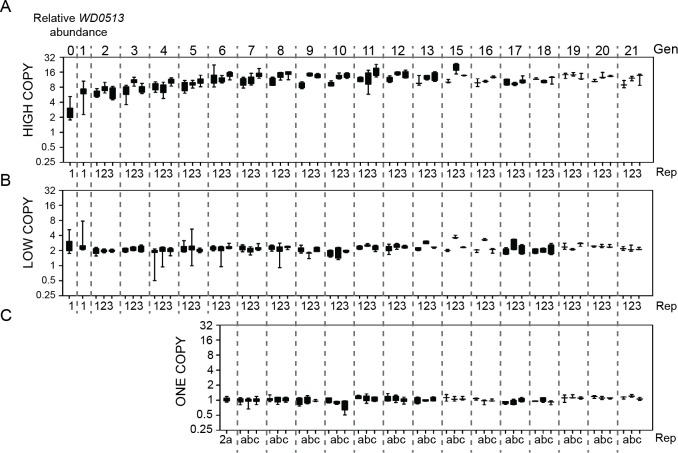
Octomom copy number is heritable and can be selected. Selection for high (A), low (B), and one (C) *WD0513* copy number in *w*MelPop in *w* ^*1118*^ flies. Selection was started with females coming from one vial (Generation zero). The female with the highest (A) and lowest (B) *WD0513* abundance was always the founder of the next generation. At generation two, both selection regimes were split into three replicate lines. At generation six, we derived a one-copy line from the low-copy selection line two that was subsequently split into three lines kept independently (C). From that point on, the low-copy regime was maintained at two Octomom copies. The boxes extend from the 25th to 75th percentile, and the whiskers include all values. Dashed lines separate generations. Supporting data can be found in S3 Data. Gen, generation; Rep, replicate.

To test Octomom amplification’s effect on *w*MelPop virulence, we established *Drosophila* lines carrying *Wolbachia* with different Octomom copy numbers. Individual females with the highest and the lowest Octomom copy number were selected throughout several generations in both *w* ^*1118*^ and *iso* backgrounds (Figs. 2 and S2). Octomom copy number is heritable: *Drosophila* mothers carrying high-copy *Wolbachia* produce mostly offspring with high-copy *Wolbachia*, while the inverse is observed for mothers with low-copy *Wolbachia*. In the course of selection for low Octomom copy number in the *w* ^*1118*^ background, we recovered a *Drosophila* line carrying *w*MelPop with only a single copy of Octomom (Fig. 2C). This single-copy Octomom line had also lost the *WD0514–WD0507* junction detected in *w*MelPop with multiple Octomom copies (S3 Fig.). Therefore, from generation six onwards, we maintained three selection regimes: high, two, and one Octomom copy number. The *w*MelPop unique synonymous SNP is present in all three selection lines, including the line carrying *Wolbachia* with a single Octomom copy (S4 Fig.).

**Fig 3:**
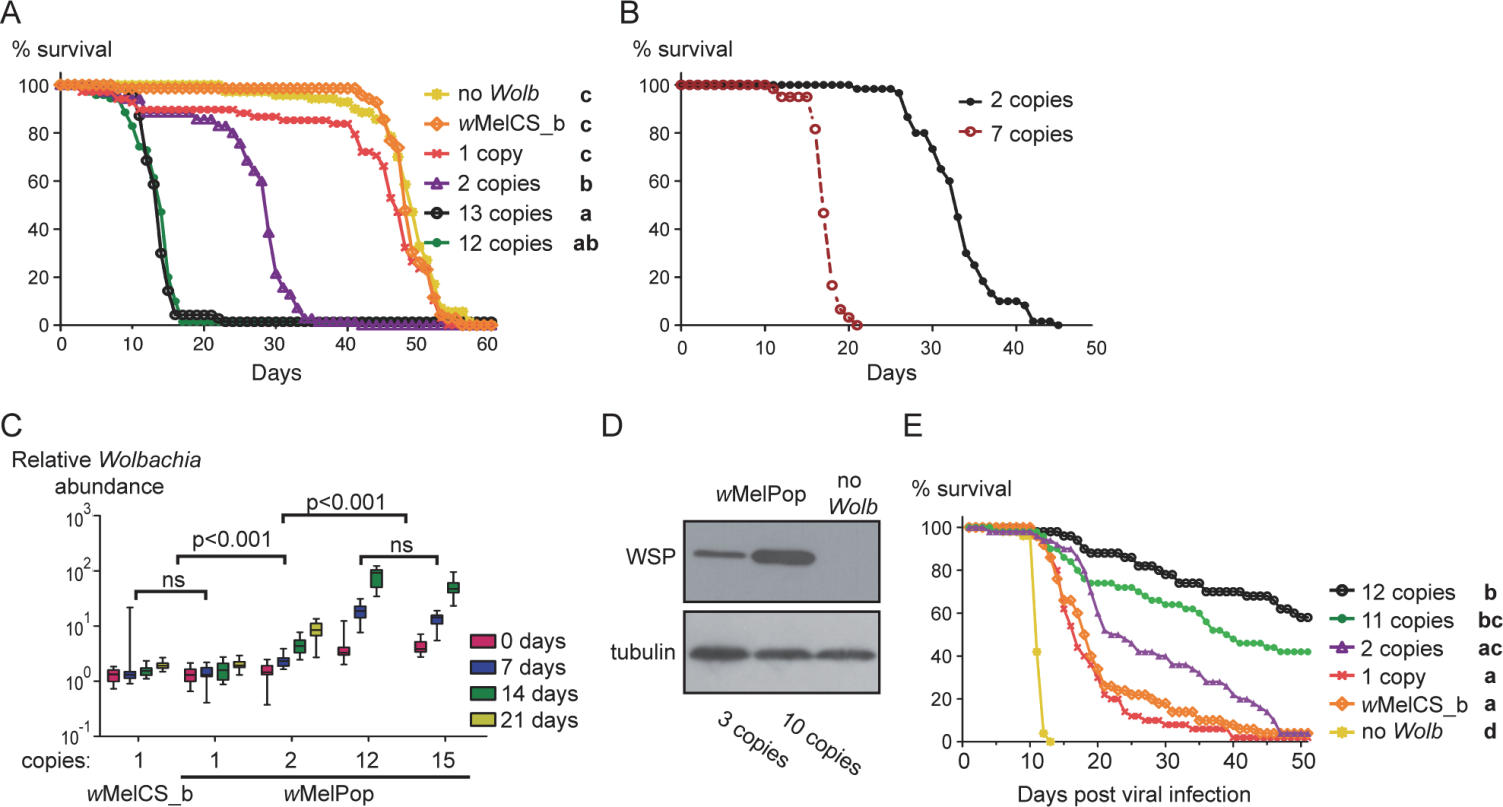
Octomom copy number determines *w*MelPop phenotypes. (A) Lifespan of female flies with different *w*MelPop Octomom copy numbers, flies with *w*MelCS_b, and *Wolbachia*-free controls at 29°C. Seventy females per line were analyzed; flies are the progeny from crosses between *iso* and *w* ^*1118*^ lines. Bold letters on the right indicate groups with significantly different survival curves by Tukey’s test of all pairwise comparisons of Cox hazard ratios. Supporting data can be found in S4 Data. (B) Lifespan of female flies from the forward selection *iso* low-copy line two (two Octomom copies) and the matched reverse selection line (seven copies) at 25°C. Mixed effects Cox model fit, *p* < 0.001. Supporting data can be found in S5 Data. (C) Time-course analysis of *Wolbachia* densities in female flies with different *w*MelPop Octomom copy numbers, starting at eclosion (day zero). Each bar represents *wsp* genomic levels in 16–20 single females (progeny from crosses between *iso* and *w* ^*1118*^ lines). The boxes extend from the 25th to 75th percentile, and the whiskers include all values. Statistical analysis was performed using a log-linear model, and the *p-*values refer to comparisons of slopes. Supporting data can be found in S6 Data. ns, non-significant. (D) Western blot with anti-WSP antibody of pools of ten 10-d-old *iso* female flies with three or ten Octomom copies. *Drosophila* tubulin was used as a loading control. (E) Survival of female flies with different *w*MelPop Octomom copy numbers upon viral infection at 18°C. Fifty females per line were analyzed; flies are the progeny from crosses between *iso* and *w* ^*1118*^ lines. Bold letters on the right indicate groups with significantly different survival curves by Tukey’s test of all pairwise comparisons of Cox hazard ratios. Supporting data can be found in S7 Data.

Taking advantage of the different selection lines, we compared the phenotypes of flies with *w*MelPop with different Octomom copy numbers. We predicted that the higher the copy number, the more severe the pathogenic phenotype, and that the one-copy Octomom line would be phenotypically identical to *w*MelCS_b. To perform these assays, we used the progeny of females individually tested for Octomom copy number. As *Wolbachia w*MelCS_b was associated with the *iso* fly genetic background and the one-copy Octomom line appeared only in the *w* ^*1118*^ background, we used hybrids between *iso* and *w* ^*1118*^ to directly compare the two (S1 and S2 Tables). All female hybrids resulting from these crosses have the same host genetic background (heterozygous between *iso* and *w* ^*1118*^) and differ in the *Wolbachia* inherited from the mother. Two high-copy Octomom lines, one in each genetic background, were used to control for potential host-genotype-specific maternal effects. Survival data demonstrate that differences in Octomom copy number lead to differences in host longevity: the more Octomom copies, the earlier the flies die (Figs. 3A and S5A–G). The line with one Octomom copy derived from *w*MelPop is indistinguishable from *w*MelCS_b and *Wolbachia*-free control (Figs. 3A and S5E–G). Even a single duplication of this region is enough to significantly shorten the host lifespan (median time to death is reduced by 39%) (Figs. 3A and S5E–G). The lifespan of flies from the two high-copy Octomom lines is further reduced, and there is no difference between these two lines (Figs. 3A and S5E–G). To further test the dependence of the phenotype on Octomom copy number, we reversed the direction of the selection in selected *iso* lines (choosing females with *w*MelPop with the highest Octomom copy number from the low-copy lines and with the lowest copy number from the high-copy lines, from generation 17 onwards) (S6A Fig.), simultaneously maintaining the forward selection regime as controls (S2 Fig.). Comparison of the lifespans of females from the forward and reverse selections confirmed that *Wolbachia* Octomom copy number determines *w*MelPop pathogenicity (Figs. 3B and S6B–D). Overall, Octomom copy number negatively correlates with longevity (S7 Fig.), and by manipulating copy number we can control *Wolbachia* virulence.

We next asked whether *Wolbachia* growth is associated with Octomom copy number. We tested *Wolbachia* levels in flies carrying *Wolbachia* with different Octomom copy numbers over time by real-time quantitative PCR (qPCR) (Fig. 3C). The higher the Octomom copy number, the higher the density of *Wolbachia*. The levels are different at eclosion, and the growth of *Wolbachia* is faster in flies with higher Octomom copy number. Both high-copy lines have the same *Wolbachia* growth rate, which is higher than the *Wolbachia* growth rate of the two-copy line. This growth rate, in turn, is higher than that of one-copy *w*MelPop and *w*MelCS_b, which have the same *Wolbachia* growth rate (Fig. 3C). We confirmed this effect of Octomom copy number on *Wolbachia* densities by comparing *Wolbachia* WSP protein abundance between flies harboring *w*MelPop with three versus ten Octomom copies (Fig. 3D). Flies carrying *Wolbachia* with ten Octomom copies had more WSP protein than flies harboring *Wolbachia* with three copies.

The density of *Wolbachia* is known to be related with *Wolbachia*-conferred antiviral protection, and *w*MelPop provides very strong protection [8,17,18,26,38–40]. This protective effect is best analyzed when flies are kept at 18°C, the temperature at which *w*MelPop is not pathogenic [41]. In flies that are raised from egg to adult at 25°C, *Wolbachia* levels at the time of infection are still related to Octomom copy number (see Fig. 3C). The survival of virus-infected flies confirmed that the higher the Octomom copy number, the stronger the antiviral protection (Figs. 3E and S5H). As with pathogenicity and growth rate, *w*MelPop with one Octomom copy is phenotypically identical to *w*MelCS_b in terms of antiviral protection.

We showed that Octomom copy number can change rapidly under direct selection (Figs. 2 and S2). Next we questioned whether Octomom copy number would be stable if this selection were relaxed. We observed that releasing our lines from copy number selection and maintaining them at 25°C in crowded vials for five generations caused a decrease in copy number in three out of four lines tested (S8 Fig.). The only line where the copy number did not change over the five generations started with two Octomom copies. Also, examination of another *w*MelPop stock did not show the expected life-shortening phenotype and, accordingly, Octomom amplification (S9 Fig.). Presumably, Octomom copy number reverted to one copy, and the phenotype was lost in this stock. All these results demonstrate that *w*MelPop *Wolbachia* is genetically and, consequently, phenotypically unstable.

**Fig 4:**
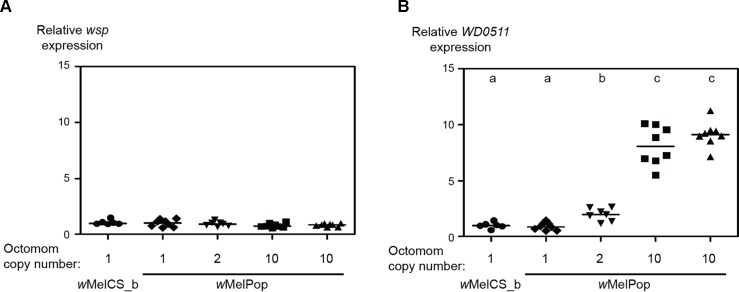
Octomom copy number determines expression of Octomom gene *WD0511*. Expression of *wsp* (outside the Octomom region) (A) and *WD0511* (within the Octomom region) (B) in flies carrying *w*MelPop with different Octomom copy numbers or carrying *w*MelCS_b. *WD0511* is differentially expressed between the lines (Tukey’s test on linear model, *p* < 0.002), except between *w*MelCS_b and one-copy Octomom *w*MelPop and between the two high-copy Octomom *w*MelPop lines. Letters indicate groups with significantly different expression levels by Tukey’s test of all pairwise comparisons of the linear model fit. There is no significant difference in the expression of *wsp* between any of the lines. All flies are hybrids between *iso* and *w* ^*1118*^ genetic background. Hybrids represented by circles and triangles are derived from *w*MelCS_b or *w*MelPop *iso* mothers, while hybrids represented by diamonds, inverted triangles, and squares are derived from *w*MelPop *w* ^*1118*^ mothers. Relative expression for each gene is calculated using *gmk* as a reference gene and is relative to that of the *w*MelCS_b samples. RNA was extracted from eight samples of ten 3- to 6-d-old *iso* males, and real-time qPCR was performed on cDNA with specific primers. Lines are medians of the replicates. Supporting data can be found in S8 Data.

Octomom amplification could promote *w*MelPop virulence in several ways, including via local or overall gene expression deregulation. The most parsimonious explanation, however, is that Octomom genes are overexpressed and that this causes the phenotype. Thus, we checked the expression of Octomom genes, immediately adjacent genes, and genes distant from the region by reverse transcription real-time qPCR. All Octomom genes, except *WD0514*, had a statistically significant higher expression in *w*MelPop than in *w*MelCS_b, but immediately adjacent genes did not (S10 Fig.). Moreover, analysis of one Octomom gene (*WD0511*) showed that expression level was dependent on *w*MelPop Octomom copy number (Fig. 4).

## Discussion

Here we identify the genetic basis of *Wolbachia w*MelPop virulence. By selecting for *Wolbachia* with different Octomom copy numbers, we show a functional link between copy number and *w*MelPop phenotypes. The more copies of Octomom, the higher the densities of *Wolbachia*, and the faster the hosts die, but the stronger the antiviral protection. The evidence we provide is stronger than a simple correlation because we are controlling Octomom copy number and determining its effect. Furthermore, all *w*MelPop phenotypes are reverted in the line selected for one Octomom copy, establishing that Octomom copy number drives these phenotypes. There is evidence that *Wolbachia* levels determine the strength of the *Wolbachia*-associated phenotypes [8,17,18,26,38–40]. Therefore, the different replication capacities of *w*MelPop variants with distinct Octomom copy numbers are the likely cause of the differences in the other phenotypes.

Woolfit and colleagues also identified Octomom amplification in the *D*. *melanogaster w*MelPop genome and a deletion of the Octomom region in a mosquito-adapted *w*MelPop variant, *w*MelPop-PGYP [62]. As *w*MelPop-PGYP retained a strong life-shortening effect in *A*. *aegypti*, while an *A*. *aegypti*–adapted *w*Mel variant was benign, the authors dismissed Octomom as responsible for the high virulence of *w*MelPop also in *D*. *melanogaster*. We argue that the difference between *w*MelPop-PGYP and *w*Mel phenotypes in mosquitoes may be due to other genetic changes accumulated during their adaptation to a new host, some already described for *w*MelPop-PGYP [62]. This phenotypic difference may also exist because *w*Mel and *w*MelPop belong to the two different monophyletic groups of *Wolbachia* from *D*. *melanogaster*: *w*Mel group and *w*MelCS group [8,68–70]. *w*MelCS-like variants replicate faster than *w*Mel-like variants and sometimes shorten the lifespan of their natural host [8], and this difference may be exacerbated in mosquitoes. Relatedly, some *Wolbachia* bacteria transinfected into a new host species induce new pathogenic phenotypes [18,71–73].

Amplification of Octomom is in agreement with common gene amplification by nonequal recombination in bacteria [64]: (i) Octomom is flanked by direct repeats (see [8,62]), (ii) it seems to amplify as a unit, since different Octomom genes are equally amplified in the same fly (Figs. 1B and S1A), (iii) we confirmed the predicted novel joint point (Figs. 1C and S3; S1 Text), and (iv) the amplification is unstable.

The degree of Octomom amplification, and the associated strength of the phenotypes, can rapidly change and is fully reversible. This shows that *Wolbachia* can evolve rapidly, and adds to the understanding of genome evolution of endosymbionts. Many endosymbionts have evolutionarily dynamic genomes [1,74]. Genomes of *Wolbachia* and other endosymbionts (including *Hamiltonella defensa*, *Serratia symbiotica*, *Sarocladium oryzae* principal endosymbiont [SOPE], and *Portiera*) are rich in mobile elements, prophages or phage-derived regions, and other repetitive DNA sequences [1,59,74–79]. These DNA elements may mobilize, amplify, or reduce in numbers, leading to genomic changes, but they can also mediate recombination and other genomic rearrangements. Comparative genomics of some closely related endosymbionts show extensive genomic rearrangements [75,76,78,80–85]. The same repetitive DNA elements may serve as a basis for gene amplification, as observed for Octomom. Consequently, gene copy number variation may be a common feature in these endosymbionts and may promote fast but reversible evolutionary changes. Accordingly, gene amplifications in other *w*Mel variants [8], other *Wolbachia* strains [80,81], and the whitefly endosymbiont *Portiera* [78] have previously been reported, although without any associated phenotypes.

Genotype–phenotype links are very rarely established in endosymbionts, as many of them cannot be cultured *in vitro*. Previous examples include a point mutation in *Buchnera aphidicola* that affects thermal tolerance provided to the aphid *Acyrthosiphon pisum* [86] and the loss of a prophage in *Hamiltonella defensa*, abrogating induced protection to parasitoids in the same aphid [87]. The involvement of Octomom genes in *Wolbachia* virulence provides a unique point of entry into understanding *Wolbachia*–host interactions at the molecular level. As Octomom genes are overexpressed and may cause the phenotype, functional analysis of Octomom-encoded proteins is required to better understand the *Wolbachia*–host interaction. The Octomom region is part of the *Wolbachia* accessory genome since it is not present in all *Wolbachia* strains and shows signs of horizontal gene transfer [8,88–91]. There are genes putatively encoding mobile elements in the flanking region (*WD0506* and *WD0515*, in the direct repeats) and inside Octomom (*WD0511*). Because of its structure and associated phenotype, the Octomom region resembles bacterial pathogenicity islands [92,93]. However, the pathogenicity seems to be expressed only when the region is amplified. The functions of Octomom genes are unknown and can only be speculated about based on the sequence of predicted proteins. Proteins encoded by three genes (*WD0512*, *WD0513*, and *WD0514*) have eukaryotic protein domains or homologs in arthropods (mosquitoes and *Daphnia*) and therefore may be effector proteins that interact with the host [8,88–91]. When highly expressed, these proteins could suppress host control over the symbiont. Other genes (*WD0506–WD0511* and *WD0515*) encode proteins that may be involved in transposition, DNA replication and repair, or transcriptional regulation [8]. Overexpression of these proteins may increase *Wolbachia*’s replication rate. It is crucial to determine which of these genes are involved in the regulation of *Wolbachia* density and which structural characteristics of the Octomom region are important.

Octomom copy number instability may confound past and future analyses of *w*MelPop phenotypes. For instance, Octomom copy number variation may have contributed to changes in *w*MelPop pathogenicity over time or associated with different host species or host genetic backgrounds [41,42,94]. This instability has to be taken into consideration in future applications of *w*MelPop-transinfected mosquitoes to prevent transmission of human pathogens. In the dengue vector *A*. *aegypti* transinfected with *w*MelPop-PGYP, currently being tested in the field [44,95], Octomom copy number instability is not a factor since this region is deleted [62]. However, *w*MelPop is also being transfected to other vectors of human diseases, such as the malaria-transmitting *Anopheles gambiae* [96] and the dengue and chikungunya vector *Aedes albopictus* [97].

We have shown variation in *Wolbachia* Octomom copy number between individual hosts within a population and across time. Genetic heterogeneity within individual hosts has been previously shown at the nucleotide level in *w*Cer1 and *w*Cer2 [98]. The instability of Octomom copy number suggests that there is also a high level of heterogeneity between *Wolbachia* bacteria within individual insects. Analysis of the dynamics and consequences of heterogeneity in gene copy numbers in somatic or germline tissues may be important to understand host–endosymbiont interactions.

Vertically transmitted endosymbionts are subjected to different levels of selection. An increase in replication may confer a fitness advantage to the bacteria in intra-host competition but a disadvantage at the inter-host level, as it can have a high cost to the host and reduce symbiont transmission. A *Drosophila* line harboring *w*MelPop was most probably isolated in the laboratory because husbandry conditions buffered the cost to flies of pathogenic bacteria and because low host population numbers increased drift. Our results demonstrate that a single mutation (a duplication) can profoundly alter endosymbiont replication. This conversion of a mutualist into a pathogen by a single genomic event suggests that virulent mutations in microbial symbionts may be frequent and constantly counter-selected. Therefore, symbiont titers may be at a labile equilibrium achieved in the course of co-evolution and to a large extent selected at the level of the symbiont.

## Materials and Methods

### Fly Strains

*D*. *melanogaster w* ^*1118*^ stock with *Wolbachia w*MelPop was kindly provided by Markus Riegler and Scott O’Neill. *w*MelPop OPL stock was kindly provided by William Sullivan and Laura Serbus. Both *w*MelPop stocks are derived from Min and Benzer original stock [19]. DrosDel isogenic background (*iso*) flies with no *Wolbachia* and with *w*MelCS_b or *w*MelPop were described before [8,24,99]. The *w*MelPop and mitochondria of this DrosDel isogenic background line derive from the *w* ^*1118*^ stock [8].

### DNA Extractions

DNA was extracted from individual flies (*w*MelPop) or pools of ten flies (*w*MelCS_b controls in the selection experiments). Each fly or pool of flies was squashed in 250 μl of 0.1 M Tris HCl, 0.1 M EDTA, and 1% SDS (pH 9.0) and incubated 30 min at 70°C. Next, 35 μl of 8 M CH_3_CO_2_K was added, and samples were mixed by shaking and incubated for 30 min on ice. Samples were then centrifuged for 15 min at 13,000 rpm at 4°C, and the supernatant was diluted 100× for qPCR.

### RNA Extractions and cDNA Synthesis

For each sample, ten 3- to 6-d-old flies were pooled and homogenized with a plastic pestle in 1 ml of Trizol Reagent (Invitrogen). RNA was extracted according to manufacturer’s protocol and resuspended in 50 μl of DEPC-treated water (Ambion). RNA concentrations were determined using a NanoDrop ND-1000 Spectrophotometer. cDNA was prepared from 1 μg of total DNAse-treated RNA using Random Primers and M-MLV Reverse Transcriptase (all Promega). Primers were pre-incubated with template RNA for 5 min at 70°C. Next, the enzyme was added, and reactions were placed at 25°C for 10 min, 37°C for 60 min, and 80°C for 10 min. cDNA was diluted 100× for qPCR.

### Real-Time Quantitative PCR

The real-time qPCR reactions were carried out in the CFX384 Real-Time PCR Detection System (Bio-Rad) as described before [8]. Briefly, each of the reactions was performed with 6 μl of iQ SYBR Green Supermix (Bio-Rad), 0.5 μl of each primer (3.6 mM), and 5 μl of diluted DNA. We performed at least two technical replicates per biological sample for each set of primers. Primer sequences were described before [8]. The following thermal cycling protocol was applied: initial 2 min at 50°C, denaturation for 10 min at 95°C, followed by 40 cycles of 30 s at 95°C, 1 min at 59°C, and 30 s at 72°C. Melting curves were examined to confirm the specificity of amplified products. Ct values were obtained using Bio-Rad CFX Manager software with default threshold settings. Ct values were subjected to a quality check—samples with standard deviation between technical replicates exceeding one were discarded. Relative amounts of transcripts and genes were calculated by the Pfaffl method [100]. To apply the method, the efficiency of each of the primer pairs was predetermined in a separate experiment. For the Octomom expression data, values were normalized to *gmk* expression. For the determination of the number of genomic Octomom copies, values were normalized to the single-copy *wsp* gene. For *Wolbachia* quantification, *wsp* levels were normalized to *Drosophila Rpl32*.

### Sequencing of the *WD0514–WD0507* Junction

The *WD0514–WD0507* junction was amplified using specific primers (Link_seq_1 and Link_seq_2), and Sanger sequencing was performed with these primers and the primers annealing inside the junction (Link_seq_3–7) by Source Bioscience. Primer sequences are listed in S3 Table.

### Selection Experiments

Selection for high- and low-copy Octomom *w*MelPop lines in *w* ^*1118*^ and *iso* backgrounds was initiated with females from a single vial of each background. For each background, ten single females were separated into individual vials and allowed to lay eggs for 5 d before being sacrificed for determination of *Wolbachia WD0513* copy number. The offspring of the female with the highest and the lowest Octomom copy number was used to start the next generation. This general procedure was repeated at every generation of selection. Three replicates of high- and low-copy Octomom selection lines for each background were established at generation two. From that point on, we selected one female/line/generation with the desired Octomom copy number (based on real-time qPCR). Female age for egg laying (0–2 d) and qPCR (5–7 d) was controlled from generation four and two for the *iso* and *w* ^*1118*^ lines, respectively. At generation seven of the *w* ^*1118*^ lines, we started to also select for one-copy Octomom *w*MelPop. At this point we selected the female with *Wolbachia* with *WD0513* copy number closest to one for this selection regime, and the female with *Wolbachia* with *WD0513* copy number closest to two for the low-copy Octomom lines.

From generation two to generation 13 of the *w* ^*1118*^ selection lines and from generation two to generation 22 of the *iso* selection lines, we were selecting from among six to ten females. From these generations on, we selected from three females per line.

At generation 14 of the *w* ^*1118*^ lines and generation 18 of the *iso* lines, the selection was not performed.

### Preparation of Flies for Phenotypic Analyses

For phenotypic analyses of flies carrying *w*MelPop with different Octomom copy numbers, single females were placed in vials, allowed to lay eggs for 5 d, and sacrificed to determine *WD0513* copy number. The progeny of females carrying *Wolbachia* with the specified Octomom copy numbers were selected for the phenotypic analyses. All lifespan assays were performed at 25°C and 29°C, the temperature regimes applied in the first report on *w*MelPop phenotypes [19].

In order to directly compare flies with *w*MelPop with the full range of Octomom copy numbers, flies with *w*MelCS_b, and flies without *Wolbachia*, we used hybrids between *w* ^*1118*^ and *iso* genetic backgrounds (S1 and S2 Tables). Females with the desired *Wolbachia* status, which is transmitted to the next generation, were crossed with males from the other genetic background. Since females were used in the phenotypic analyses, their genetic backgrounds were all equal and heterozygous between *w* ^*1118*^ and *iso*, irrespective of the direction of the crosses. The mitochondria from these two lines should be identical since they share a very recent common ancestor [8]. We used females with high Octomom copy number from both genetic backgrounds to control for the possible influence of the direction of the cross and maternal effects potentially associated with different backgrounds.

### Lifespan and *Wolbachia* Levels Experiments

Females whose mothers’ Octomom copy number was assessed by qPCR were collected at eclosion (ten per tube), allowed to mate for 24 h (five males per tube), separated from males, and either checked for survival at 25°C or 29°C every day or kept at 25°C and sacrificed at the indicated time points for *Wolbachia* density quantification. Females were maintained on a standard cornmeal diet without live yeast and were passed to fresh vials every 3 d. The mothers of females used for phenotypic analyses were derived from selection lines at the generations indicated in S2 Table.

### Virus Production and Infection

DCV was produced and titrated as described before [8,24]. Infections were performed by pricking 1- to 2-d-old female flies with virus at 10^9^ TCID_50_ (median tissue culture infectious dose)/ml. After infection, flies were kept in vials without live yeast, ten flies per vial, at 18°C. It has been shown previously that *w*MelPop is not pathogenic to the flies at this temperature [41]. Flies were checked for survival daily and passed to fresh vials every 5 d.

### Statistical Analysis

Survival data were analyzed by Cox proportional hazard mixed effects models. Octomom copy number was considered a fixed effect, and replicate tube (containing ten flies) within the same experiment was considered random. Model fitting was done using the coxme package in R [101]. Tukey´s test was applied for pairwise comparisons of Cox hazard ratios between flies with all *w*MelPop lines, flies with *w*MelCS_b, and flies without *Wolbachia*.

Analysis of growth curves of *w*MelPop lines with different Octomom copy number was performed with log-linear model fits (lm in R). The slopes of different fitted regression lines were compared and corrected for multiple comparisons (Bonferroni correction).

Spearman correlation between Octomom copy number and median time to death was performed in R (cor.test).

Comparison of the expression of several *Wolbachia* genes between *w*MelCS_b and *w*MelPop (S10 Fig.) was done with the *t*-test in R (t.test) and was corrected for multiple comparisons with the Bonferroni correction.

Comparison of *wsp* and *WD0511* gene expression between fly lines carrying different *Wolbachia* (Fig. 4) was performed with a log-linear model fit (lm in R), and the different lines were compared pairwise with a Tukey’s test.

### Western Blot

Ten mated females from high- and low-copy *iso* selection lines, whose mothers were individually tested for Octomom copy number, were aged for 10 d before protein extraction. Flies without *Wolbachia* were used as a negative control. Anti-WSP rabbit polyclonal antibody was kindly provided by Kostas Bourtzis [102,103] and pre-absorbed in fixed *Wolbachia*-free *D*. *melanogaster* embryos. Anti-beta-tubulin mouse monoclonal E7 antibody was acquired from the Developmental Studies Hybridoma Bank [104].

## Supporting Information

S1 Data Relative *WD0513* copy number in single females carrying *w*MelPop from different stocks (data for Fig. 1A). (XLSX)

S2 Data Relative *WD0507* and *WD0513* copy number from individual flies carrying *w*MelPop (data for Fig. 1B). (XLS)

S3 Data Relative *WD0513* copy number in *w*MelPop in *w* ^*1118*^ flies throughout selection (data for Fig. 2). (XLSX)

S4 Data Lifespan data for flies carrying *w*MelPop with different Octomom copy numbers (data for Fig. 3A). (XLS)

S5 Data Lifespan data for flies carrying *w*MelPop from forward and reverse selection (data for Fig. 3B). (XLS)

S6 Data Time-course analysis of relative levels of *Wolbachia w*MelPop with different Octomom copy numbers (data for Fig. 3C). (XLS)

S7 Data Survival data for DCV-infected flies carrying *w*MelPop with different Octomom copy numbers, *w*MelCS_b, or no *Wolbachia* (data for Fig. 3E). (XLS)

S8 Data Relative expression of *wsp* and *WD0511* in flies carrying *w*MelPop with different Octomom copy numbers or *w*MelCS_b (data for Fig. 4). (CSV)

S9 Data Relative *WD0507*, *WD0510*, *WD0513*, *rpoD*, and *gmk* copy numbers from individual flies carrying *w*MelPop (data for S1 Fig.). (XLS)

S10 Data Relative *WD0513* copy number in *w*MelPop in *iso* flies throughout selection (data for S2 Fig.). (XLSX)

S11 Data Lifespan data for *iso* flies carrying *w*MelPop with different Octomom copy numbers (data for S5A Fig.). (XLS)

S12 Data Lifespan data for *iso* flies carrying *w*MelPop with different Octomom copy numbers (data for S5B Fig.). (XLS)

S13 Data Lifespan data for *w* ^*1118*^ flies carrying *w*MelPop with different Octomom copy numbers (data for S5C Fig.). (XLS)

S14 Data Lifespan data for *w* ^*1118*^ flies carrying *w*MelPop with different Octomom copy numbers (data for S5D Fig.). (XLS)

S15 Data Lifespan data for flies carrying *w*MelPop with different Octomom copy numbers, *w*MelCS_b, or no *Wolbachia* (data for S5E Fig.). (XLS)

S16 Data Lifespan data for flies carrying *w*MelPop with different Octomom copy numbers, *w*MelCS_b, or no *Wolbachia* (data for S5F Fig.). (XLS)

S17 Data Lifespan data for flies carrying *w*MelPop with different Octomom copy numbers, *w*MelCS_b, or no *Wolbachia* (data for S5G Fig.). (XLS)

S18 Data Survival data of DCV-infected flies carrying *w*MelPop with different Octomom copy numbers, *w*MelCS_b, or no *Wolbachia* (data for S5H Fig.). (XLS)

S19 Data Relative *WD0513* copy number in *iso* flies carrying *w*MelPop throughout reverse selection (data for S6A Fig.). (XLSX)

S20 Data Lifespan data for flies carrying *w*MelPop from forward and reverse selection (data for S6B Fig.). (XLS)

S21 Data Lifespan data for flies carrying *w*MelPop from forward and reverse selection (data for S6C Fig.). (XLS)

S22 Data Lifespan data for flies carrying *w*MelPop from forward and reverse selection (data for S6D Fig.). (XLS)

S23 Data Median time to death and Octomom copy number in experiments shown in Figs. 3A and S5A–G (data for S7 Fig.). (XLS)

S24 Data Relative *WD0513* copy number in flies carrying *w*MelPop in the absence of selection (data for S8 Fig.). (XLSX)

S25 Data Relative *WD0513* copy number in single females carrying *w*MelPop from different stocks (data for S9A Fig.). (XLSX)

S26 Data Lifespan data for flies carrying *w*MelPop OPL, *w*MelCS_b, or no *Wolbachia* (data for S9B Fig.). (XLS)

S27 Data Relative expression of Octomom genes and other *Wolbachia* genes in flies carrying *w*MelCS_b or *w*MelPop (data for S10 Fig.). (XLS)

S1 Fig Different Octomom genes are amplified to the same extent in individual *w*MelPop flies. Octomom gene copy number variability relative to *wsp* between *w*MelPop *iso* flies. qPCR was performed on DNA from single females from *iso* line three (Fig. 1A) for *WD0507*, *WD0510*, and *WD0513* (A) and *rpoD* and *gmk* (B). *w*MelCS_b flies were used for copy number normalization. Supporting data can be found in S9 Data.
(TIF)

S2 Fig Selection for *w*MelPop with high and low Octomom copy number in *iso* flies. The bars for generation zero correspond to the data for *iso* line three from Fig. 1A. The female with the highest or lowest *WD0513* copy number was always the founder of the next generation. After the first generation, three females with high and low copy number gave rise to three replicate lines that were maintained separately for the subsequent generations. The boxes extend from the 25th to 75th percentile, and the whiskers include all values. Dashed lines separate the generations. Gen, generation; Rep, replicate. Supporting data can be found in S10 Data.
(TIF)

S3 Fig PCR of the predicted *WD0514–WD0507* junction in flies harboring *w*MelPop with a single Octomom copy. *w*MelCS_b was used as a negative control, and *w*MelPop with two and ten Octomom copies were used as positive controls for the *WD0514–WD0507* junction. Flies without *Wolbachia* (*iso*) were used as a negative control for *wsp*. Two samples of each *Wolbachia* variant were used.
(TIF)

S4 Fig Alignment of the sequences containing the *w*MelPop unique SNP site from *w*MelCS_b and *w*MelPop selection lines with one, two and a high number of Octomom copies. CLUSTAL O (1.2.1) multiple sequence alignment [105–107] was used to align the sequences surrounding the *w*MelPop unique SNP at position 943,443 in the *w* ^*1118*^ selection lines. Position 943,443 is highlighted in yellow.
(TIF)

S5 Fig Octomom copy number determines *w*MelPop phenotypes. (A and B) One hundred *iso* females from high- and low-copy selection regimes were checked for survival at 25°C every day. Mixed effects Cox model fit, high versus low copy number for both replicates, *p* < 0.001. Supporting data can be found in S11 and S12 Data. (C and D) One hundred *w* ^*1118*^ females from high- and low-copy selection regimes were checked for survival at 25°C (C) or 29°C (D) every day. Mixed effects Cox model fit, high versus low copy number at both temperatures, *p* < 0.001. Supporting data can be found in S13 and S14 Data. (E–G) Sixty–seventy females carrying *w*MelPop with different Octomom copy numbers were monitored daily for survival at 29°C (E) or at 25°C (F and G). Females are the progeny from crosses between *iso* and *w* ^*1118*^ lines. Letters refer to groups with significantly different survival curves according to Tukey’s test of all pairwise comparisons of Cox hazard ratios. The experiment at 29°C is a replicate of the one presented in Fig. 3A. Supporting data can be found in S15–S17 Data. (H) One hundred females with different *w*MelPop Octomom copy numbers were pricked with DCV (10^9^ TCID_50_/ml), and survival was followed daily. Females are the progeny from crosses between *iso* and *w* ^*1118*^ lines. Letters refer to groups with significantly different survival curves according to Tukey’s test of all pairwise comparisons of Cox hazard ratios. This experiment is a replicate of the one shown in Fig. 3E. Supporting data can be found in S18 Data.
(TIF)

S6 Fig Phenotypic responses to reverse selection. (A) At generation 17 of the selection for *w*MelPop *iso* lines with high and low *WD0513* copy number (S2 Fig.), the selection was reversed. This reverse selection was performed in all three replicate lines from the high- and low-copy selection regimes by selecting the female with the highest *WD0513* abundance from each low-copy line and the female with the lowest *WD0513* abundance from each high-copy line (forward selection also continued, as shown in S2 Fig.). The boxes extend from the 25th to 75th percentile, and the whiskers include all values. Dashed lines separate the generations. Gen, generation; Rep, replicate. Supporting data can be found in S19 Data. (B and C) Lifespan of females of reversely selected high-copy lines was compared with that of high-copy females under forward selection at generation 22. Fifty females per line were used. (B) High-copy line one (nine Octomom copies) versus reverse high-copy line one (five copies) (C) High-copy line three (ten copies) versus reverse high-copy line three (six copies). Tukey’s test on the mixed effects Cox model fit, high versus low copy number, *p* < 0.001 and *p* = 0.0321 for lines one and three, respectively. Supporting data can be found in S20 and S21 Data. (D) Lifespan of females from forward selection low-copy line three (3.5 Octomom copies) and the corresponding reverse selection line (eight copies) at generation 22. Fifty females per line were used. Tukey’s test on the mixed effects Cox model fit, high versus low copy number, *p* < 0.001. Supporting data can be found in S22 Data.
(TIF)

S7 Fig Negative correlation between Octomom copy number and host longevity. Median time to death (days) for lifespan experiments performed (Figs. 3A and S5A–G) is plotted as a function of Octomom copy number (relative *WD0513* copy number). These data refer to flies with two different genetic backgrounds and experiments performed at two different temperatures. The two variables are negatively correlated (Spearman correlation *rho* = −0.701, *p* < 0.001). Supporting data can be found in S23 Data.
(TIF)

S8 Fig Release of selection pressure leads to a change in Octomom copy number. Selection was released in *w*MelPop *iso* flies at generation 26. The progeny of single females from generation 26 were kept without selection for Octomom copy number for five generations by passing all the flies to a new tube every 20 d. After these five generations, ten females per line were scored for *WD0513* copy number in their *Wolbachia* bacteria. Plotted are the original selection lines at generation 26, the same selected lines at generation 31 (the high-copy-number line was selected for ten Octomom copies from generation 29 onwards), and released selection lines at generation 31. The mothers of selected lines are represented by triangular data points, the mothers of the released selection lines are represented by blue circular data points. Lines are medians of the points at each generation/treatment. Octomom copy number decreased in three out of four lines released from selection. The only line that did not show a decrease started with two copies of Octomom. Supporting data can be found in S24 Data.
(TIF)

S9 Fig Lack of Octomom amplification and virulent phenotype in a different *w*MelPop stock. (A) Comparison of *WD0513* copy number within different *w*MelPop *iso* and *w* ^*1118*^ stocks kept in the Teixeira lab (Fig. 1A) with *w*MelPop stock obtained from another lab (*w*MelPop OPL [original Popcorn line]). DNA from single females was extracted for qPCR. *w*MelCS_b *iso* flies were used for copy number normalization, and *wsp* was used as a reference gene. Lines are medians of the replicates. Supporting data can be found in S25 Data. (B) Lifespan of females without *Wolbachia*, with *w*MelCS_b, and with *w*MelPop OPL. Females are the progeny from crosses between flies of the *iso* and the *w*MelPop OPL genetic backgrounds. One hundred females were collected at eclosion, allowed to mate for 24 h, separated from males, and scored daily for survival at 29°C. Letters refer to groups with significantly different survival curves according to Tukey’s test of all pairwise comparisons of Cox hazard ratios. Supporting data can be found in S26 Data.
(TIF)

S10 Fig Octomom amplification leads to higher expression of Octomom genes. Expression of genes in the Octomom region (*WD0507–WD0514*), in the flanking repeated region (*WD0506/WD0515*), in the immediately adjacent region (*WD0505* and *WD0519*), and in other locations of the chromosome (*wsp* and *rpoD*) in *w*MelCS_b (A) and *w*MelPop (B) (both in DrosDel isogenic background). The expression levels of *WD0506–WD0513* are higher in *w*MelPop than in *w*MelCS_b (*t*-test, *p* < 0.001 for all). The expression levels of Octomom gene *WD0514* and genes outside Octomom (*wsp*, *rpoD*, *WD0505*, and *WD0519*) are not significantly different between the two *Wolbachia* variants. Relative expression for each gene is calculated using *gmk* as a reference gene and is relative to that of *w*MelCS_b samples. RNA was extracted from eight samples of ten 3- to 6-d-old *iso* males, and real-time qPCR was performed on cDNA with specific primers. Lines are medians of the replicates. Cycle threshold values for the genes *WD0507*, *WD0513*, and *WD0514* are high, indicating low gene expression levels for these genes. These cycle threshold values fall in a nonlinear section of the standard curve, making the quantification inaccurate. Moreover, cycle threshold values for some reactions were below the detection limit. Supporting data can be found in S27 Data.
(TIF)

S1 Table Genetic background of females used in reciprocal crosses to generate *w* ^*1118*^ *× iso* hybrids (Figs. 3A, 3C, 3E, 4, and S5E–H). (DOCX)

S2 Table Selection generation number origin of mothers of the flies used for phenotypic analyses. (DOCX)

S3 Table Oligonucleotide primers used for amplification and sequencing of the *WD0514–WD0507* junction. (DOCX)

S1 Text Sequence of the new *WD0514–WD0507* junction. The sequencing of the PCR band (Fig. 1C) was performed with primers Link_seq_1–7 (S3 Table).
(TXT)

## Abbreviations:

DCV: *Drosophila* C virus
qPCR: quantitative PCR
