# Supplementary figures and images for: Mutualism Breakdown by Amplification of Wolbachia Genes
Source: PLoS Biol. 2015 Feb 10;13(2):e1002065. doi: 10.1371/journal.pbio.1002065 (PMC4323108; doi:10.1371/journal.pbio.1002065)

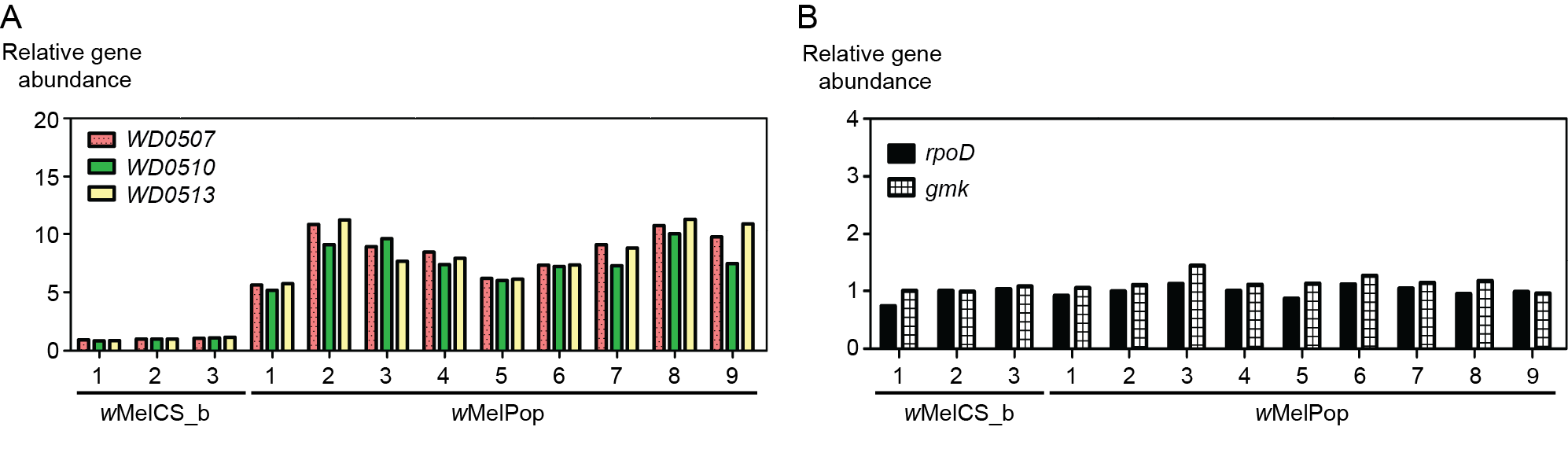

Supplement: S1 Fig — Octomom gene copy number variability relative to wsp between wMelPop iso flies. qPCR was performed on DNA from single females from iso line three (Fig. 1A) for WD0507, WD0510, and WD0513 (A) and rpoD and gmk (B). wMelCS_b flies were used for copy number normalization. Supporting data can be found in S9 Data. (TIF) [file pbio.1002065.s028.tif]

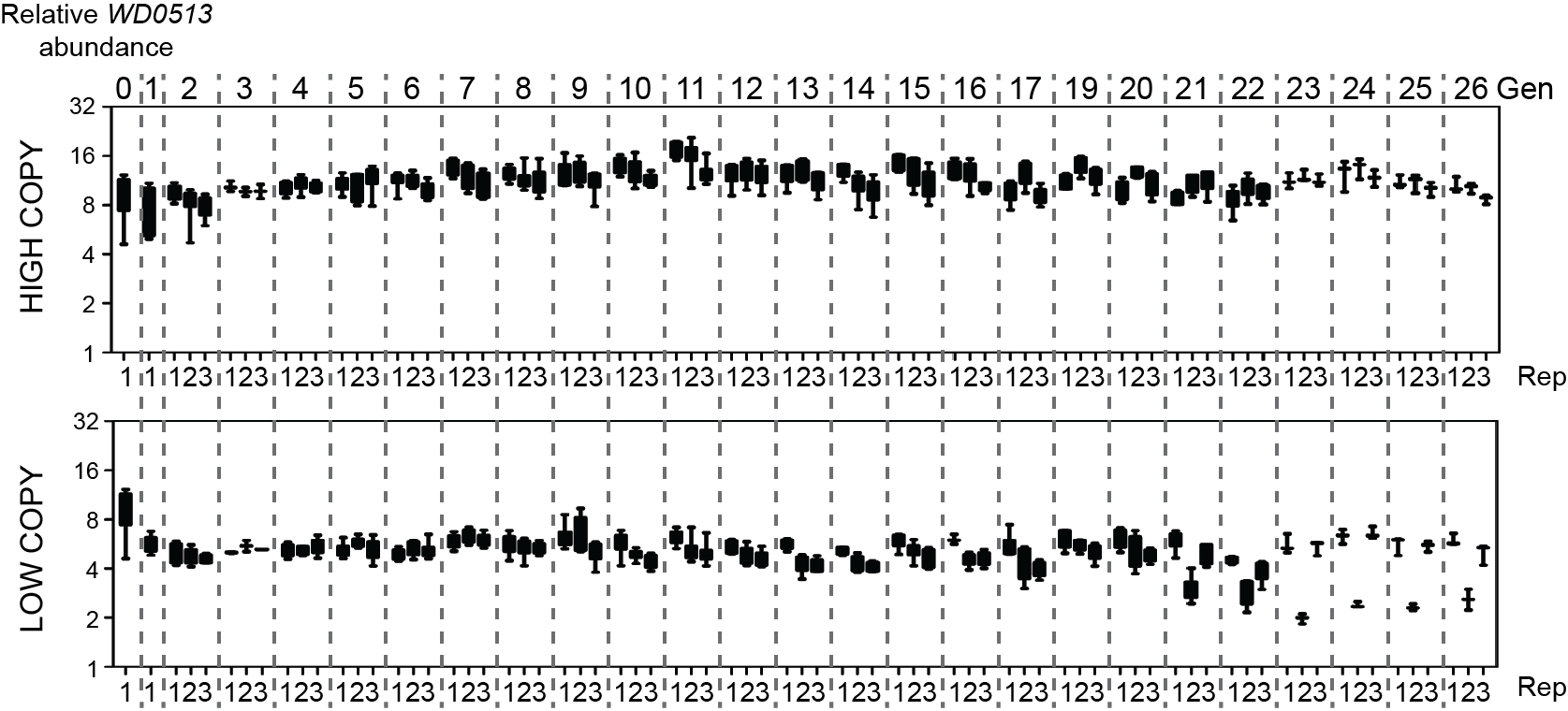

Supplement: S2 Fig — The bars for generation zero correspond to the data for iso line three from Fig. 1A. The female with the highest or lowest WD0513 copy number was always the founder of the next generation. After the first generation, three females with high and low copy number gave rise to three replicate lines that were maintained separately for the subsequent generations. The boxes extend from the 25th to 75th percentile, and the whiskers include all values. Dashed lines separate the generations. Gen, generation; Rep, replicate. Supporting data can be found in S10 Data. (TIF) [file pbio.1002065.s029.tif]

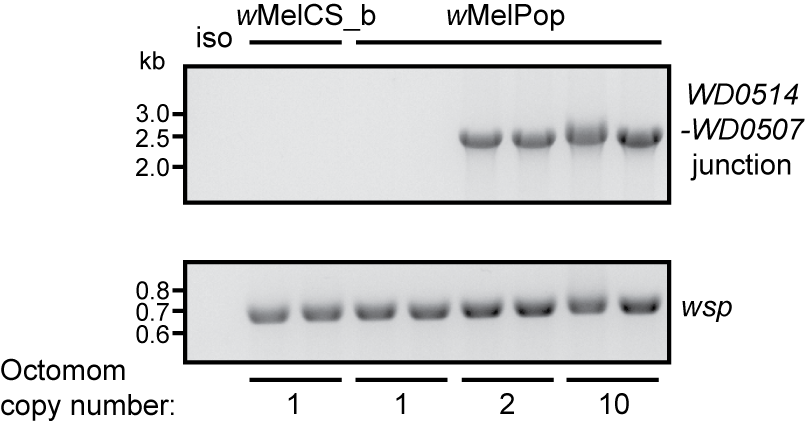

Supplement: S3 Fig — wMelCS_b was used as a negative control, and wMelPop with two and ten Octomom copies were used as positive controls for the WD0514–WD0507 junction. Flies without Wolbachia (iso) were used as a negative control for wsp. Two samples of each Wolbachia variant were used. (TIF) [file pbio.1002065.s030.tif]

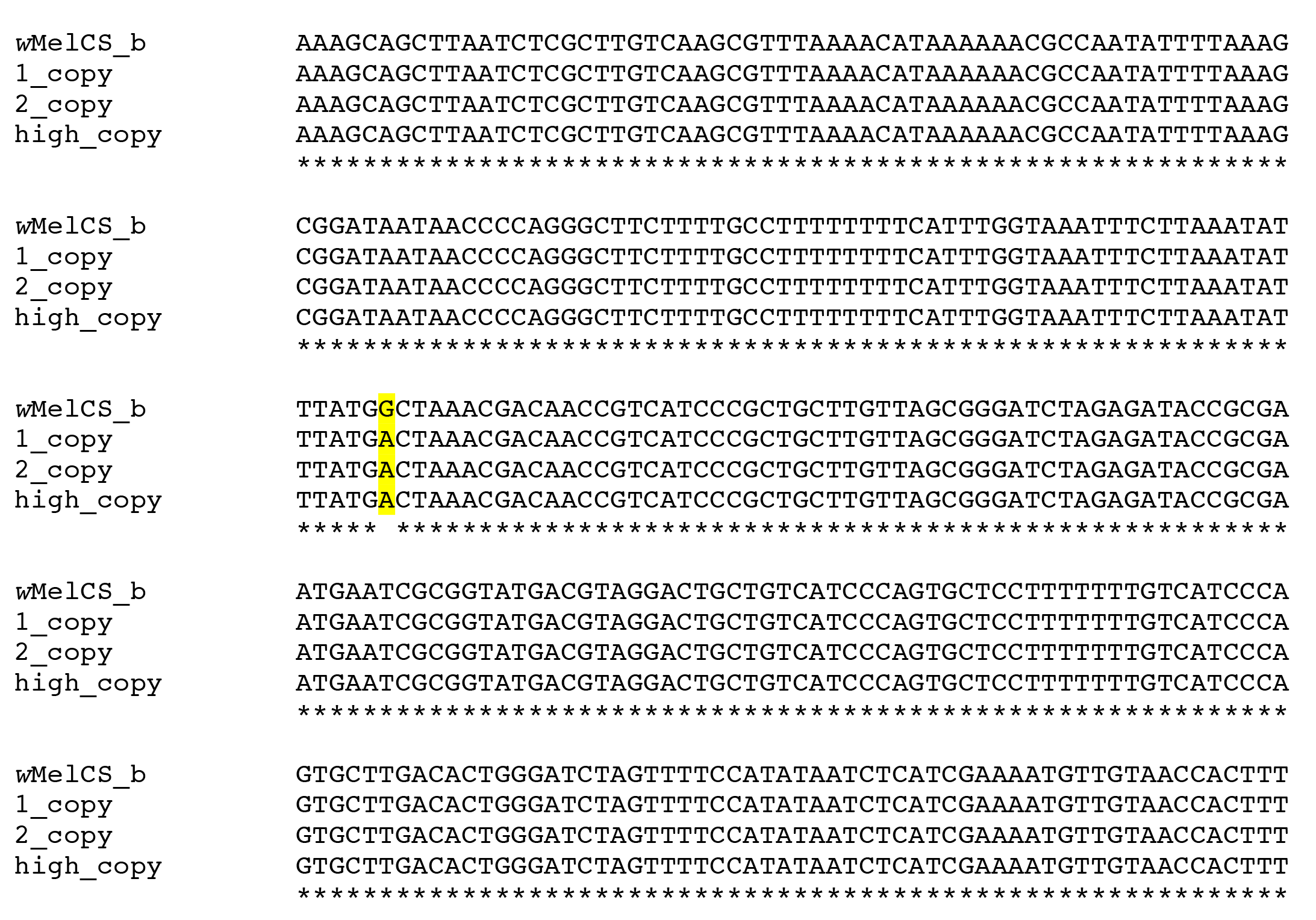

Supplement: S4 Fig — CLUSTAL O (1.2.1) multiple sequence alignment [105–107] was used to align the sequences surrounding the wMelPop unique SNP at position 943,443 in the w 1118 selection lines. Position 943,443 is highlighted in yellow. (TIF) [file pbio.1002065.s031.tif]

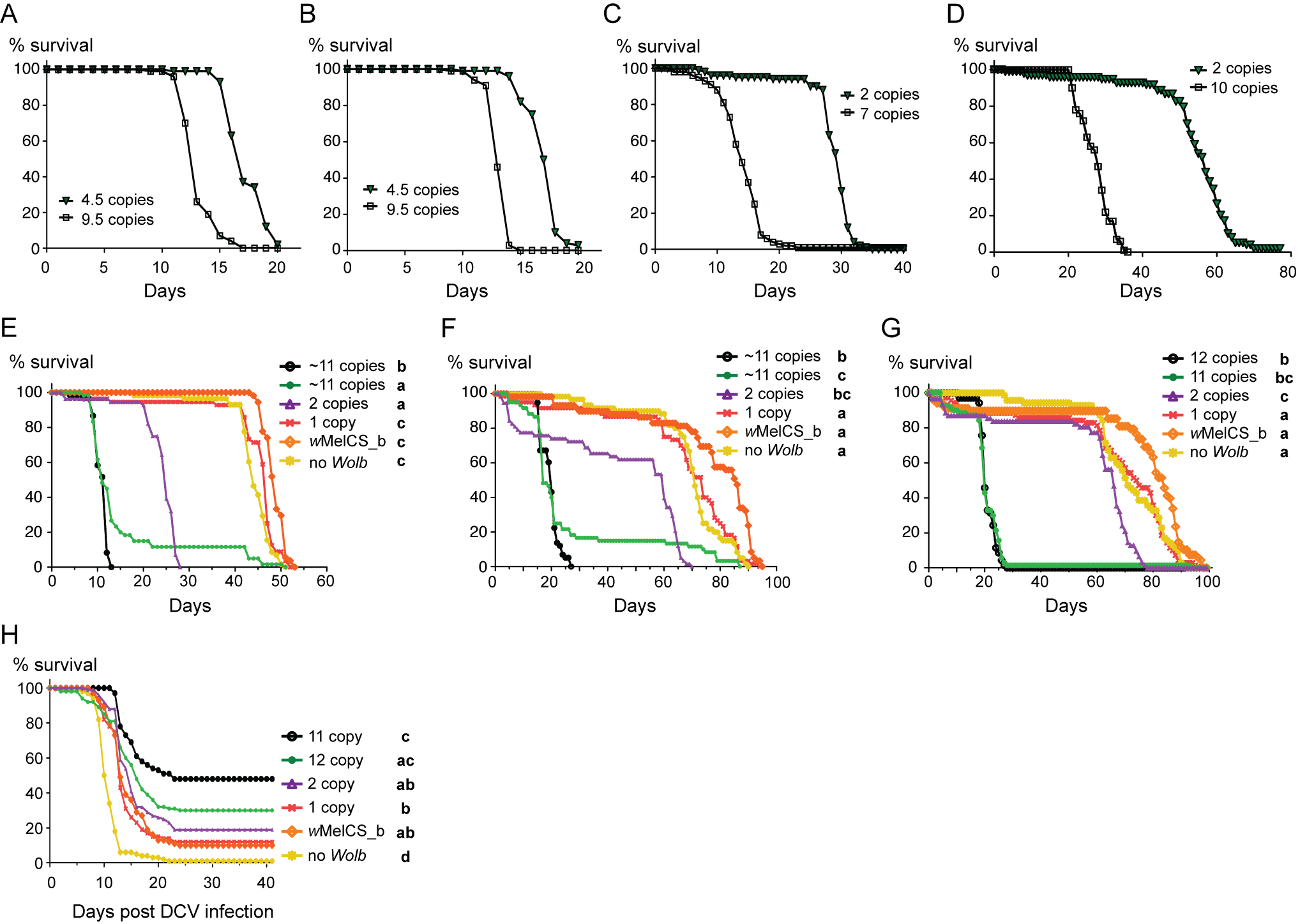

Supplement: S5 Fig — (A and B) One hundred iso females from high- and low-copy selection regimes were checked for survival at 25°C every day. Mixed effects Cox model fit, high versus low copy number for both replicates, p < 0.001. Supporting data can be found in S11 and S12 Data. (C and D) One hundred w 1118 females from high- and low-copy selection regimes were checked for survival at 25°C (C) or 29°C (D) every day. Mixed effects Cox model fit, high versus low copy number at both temperatures, p < 0.001. Supporting data can be found in S13 and S14 Data. (E–G) Sixty–seventy females carrying wMelPop with different Octomom copy numbers were monitored daily for survival at 29°C (E) or at 25°C (F and G). Females are the progeny from crosses between iso and w 1118 lines. Letters refer to groups with significantly different survival curves according to Tukey’s test of all pairwise comparisons of Cox hazard ratios. The experiment at 29°C is a replicate of the one presented in Fig. 3A. Supporting data can be found in S15–S17 Data. (H) One hundred females with different wMelPop Octomom copy numbers were pricked with DCV (109 TCID50/ml), and survival was followed daily. Females are the progeny from crosses between iso and w 1118 lines. Letters refer to groups with significantly different survival curves according to Tukey’s test of all pairwise comparisons of Cox hazard ratios. This experiment is a replicate of the one shown in Fig. 3E. Supporting data can be found in S18 Data. (TIF) [file pbio.1002065.s032.tif]

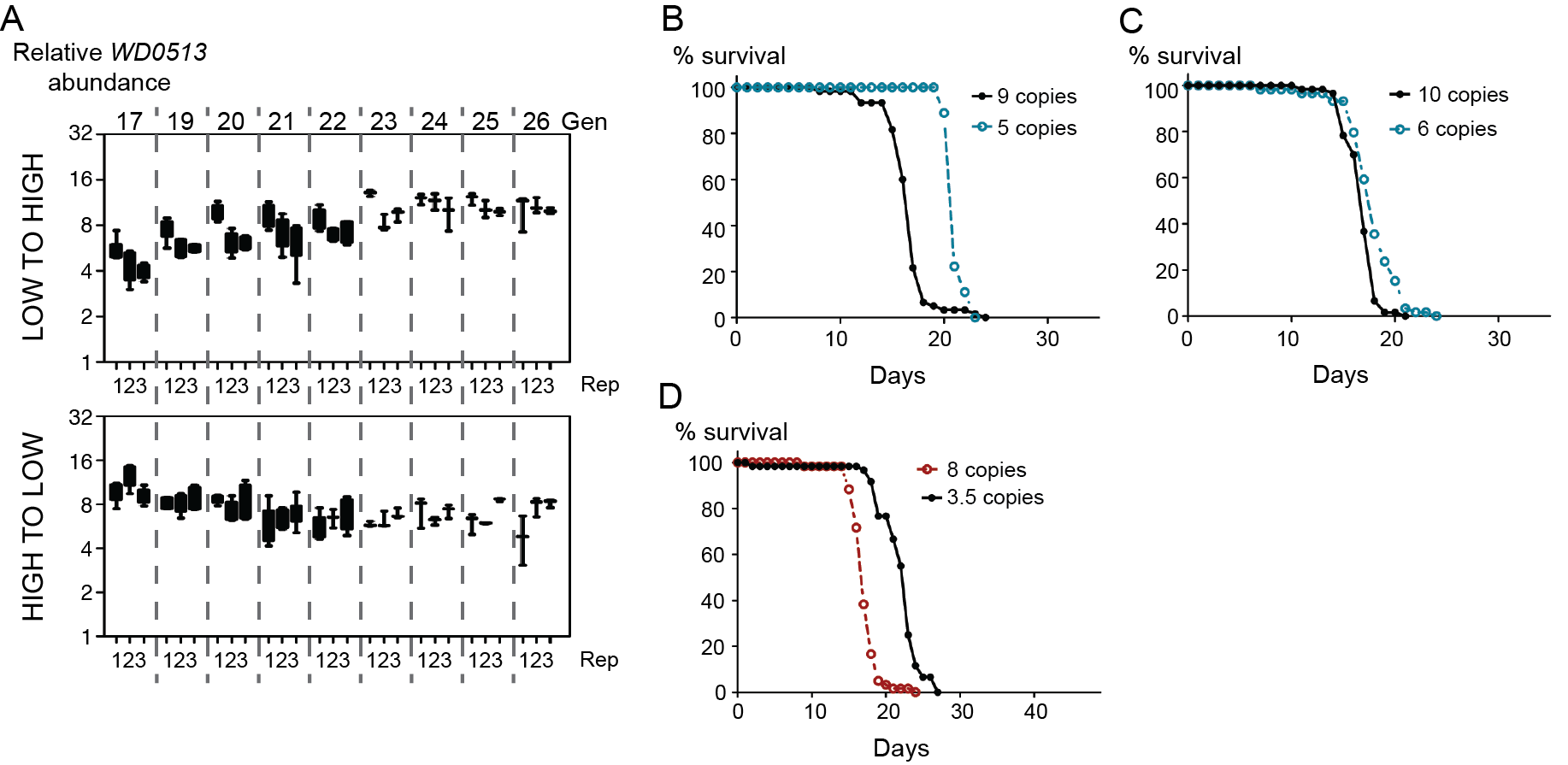

Supplement: S6 Fig — (A) At generation 17 of the selection for wMelPop iso lines with high and low WD0513 copy number (S2 Fig.), the selection was reversed. This reverse selection was performed in all three replicate lines from the high- and low-copy selection regimes by selecting the female with the highest WD0513 abundance from each low-copy line and the female with the lowest WD0513 abundance from each high-copy line (forward selection also continued, as shown in S2 Fig.). The boxes extend from the 25th to 75th percentile, and the whiskers include all values. Dashed lines separate the generations. Gen, generation; Rep, replicate. Supporting data can be found in S19 Data. (B and C) Lifespan of females of reversely selected high-copy lines was compared with that of high-copy females under forward selection at generation 22. Fifty females per line were used. (B) High-copy line one (nine Octomom copies) versus reverse high-copy line one (five copies) (C) High-copy line three (ten copies) versus reverse high-copy line three (six copies). Tukey’s test on the mixed effects Cox model fit, high versus low copy number, p < 0.001 and p = 0.0321 for lines one and three, respectively. Supporting data can be found in S20 and S21 Data. (D) Lifespan of females from forward selection low-copy line three (3.5 Octomom copies) and the corresponding reverse selection line (eight copies) at generation 22. Fifty females per line were used. Tukey’s test on the mixed effects Cox model fit, high versus low copy number, p < 0.001. Supporting data can be found in S22 Data. (TIF) [file pbio.1002065.s033.tif]

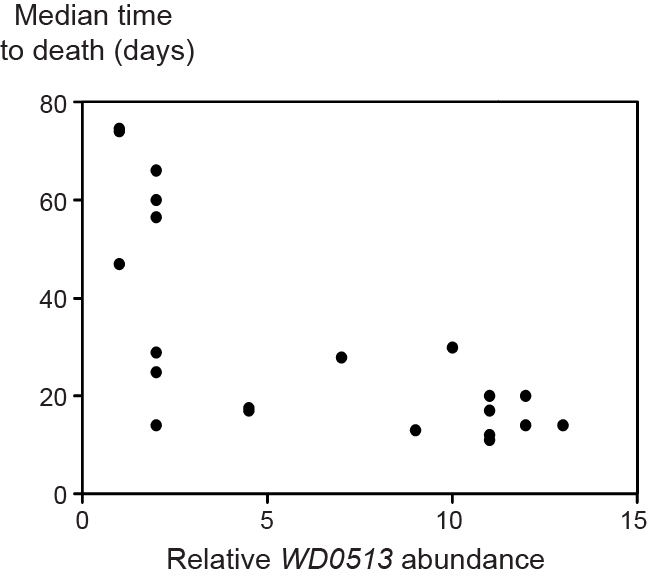

Supplement: S7 Fig — Median time to death (days) for lifespan experiments performed (Figs. 3A and S5A–G) is plotted as a function of Octomom copy number (relative WD0513 copy number). These data refer to flies with two different genetic backgrounds and experiments performed at two different temperatures. The two variables are negatively correlated (Spearman correlation rho = −0.701, p < 0.001). Supporting data can be found in S23 Data. (TIF) [file pbio.1002065.s034.tif]

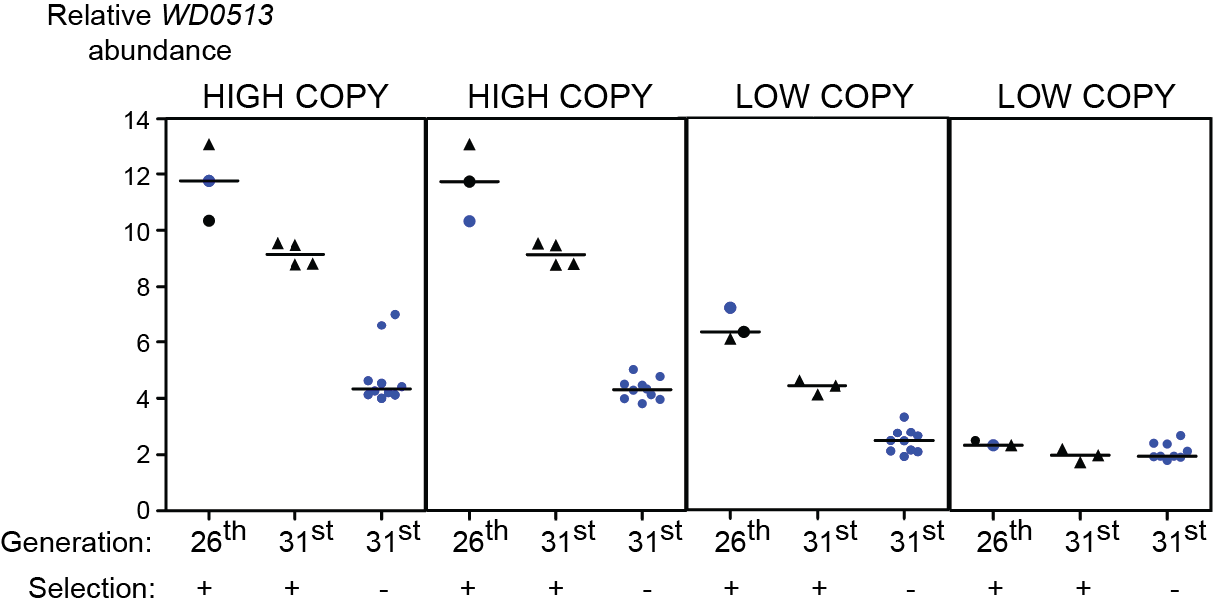

Supplement: S8 Fig — Selection was released in wMelPop iso flies at generation 26. The progeny of single females from generation 26 were kept without selection for Octomom copy number for five generations by passing all the flies to a new tube every 20 d. After these five generations, ten females per line were scored for WD0513 copy number in their Wolbachia bacteria. Plotted are the original selection lines at generation 26, the same selected lines at generation 31 (the high-copy-number line was selected for ten Octomom copies from generation 29 onwards), and released selection lines at generation 31. The mothers of selected lines are represented by triangular data points, the mothers of the released selection lines are represented by blue circular data points. Lines are medians of the points at each generation/treatment. Octomom copy number decreased in three out of four lines released from selection. The only line that did not show a decrease started with two copies of Octomom. Supporting data can be found in S24 Data. (TIF) [file pbio.1002065.s035.tif]

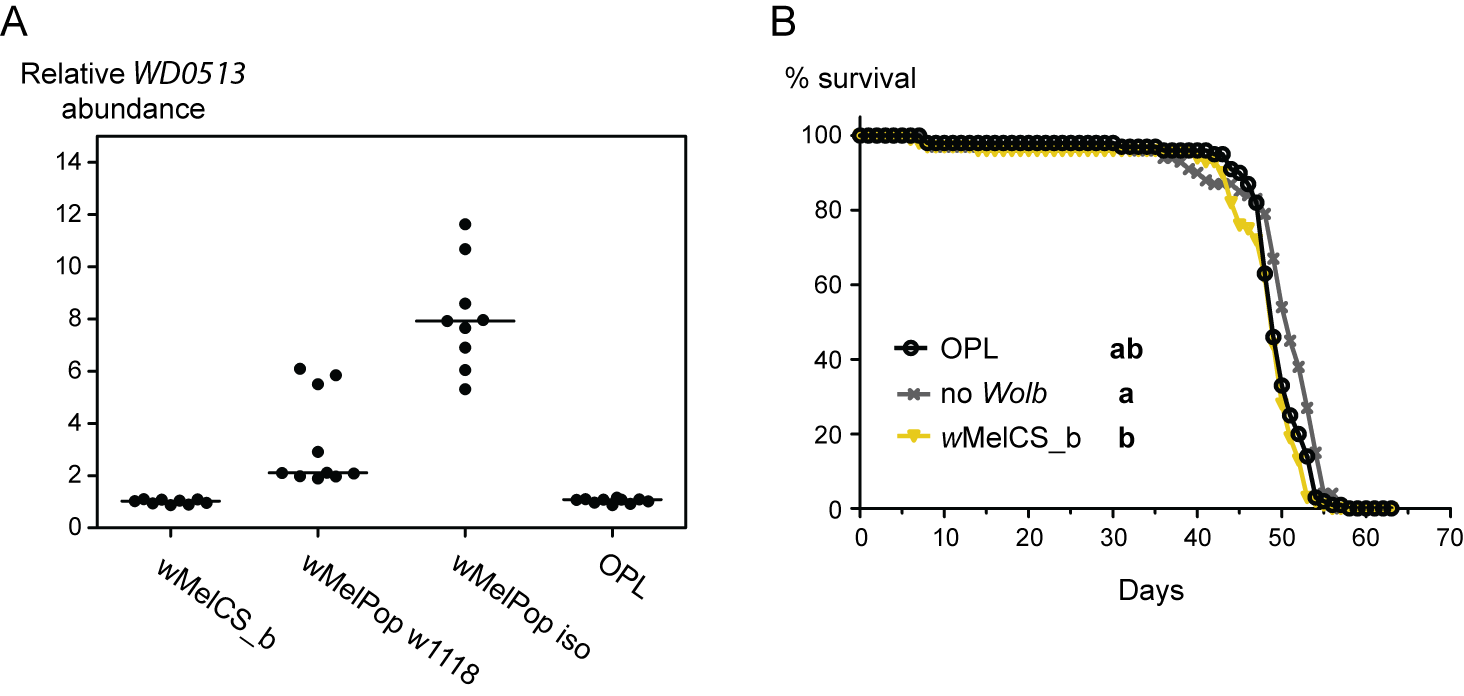

Supplement: S9 Fig — (A) Comparison of WD0513 copy number within different wMelPop iso and w 1118 stocks kept in the Teixeira lab (Fig. 1A) with wMelPop stock obtained from another lab (wMelPop OPL [original Popcorn line]). DNA from single females was extracted for qPCR. wMelCS_b iso flies were used for copy number normalization, and wsp was used as a reference gene. Lines are medians of the replicates. Supporting data can be found in S25 Data. (B) Lifespan of females without Wolbachia, with wMelCS_b, and with wMelPop OPL. Females are the progeny from crosses between flies of the iso and the wMelPop OPL genetic backgrounds. One hundred females were collected at eclosion, allowed to mate for 24 h, separated from males, and scored daily for survival at 29°C. Letters refer to groups with significantly different survival curves according to Tukey’s test of all pairwise comparisons of Cox hazard ratios. Supporting data can be found in S26 Data. (TIF) [file pbio.1002065.s036.tif]

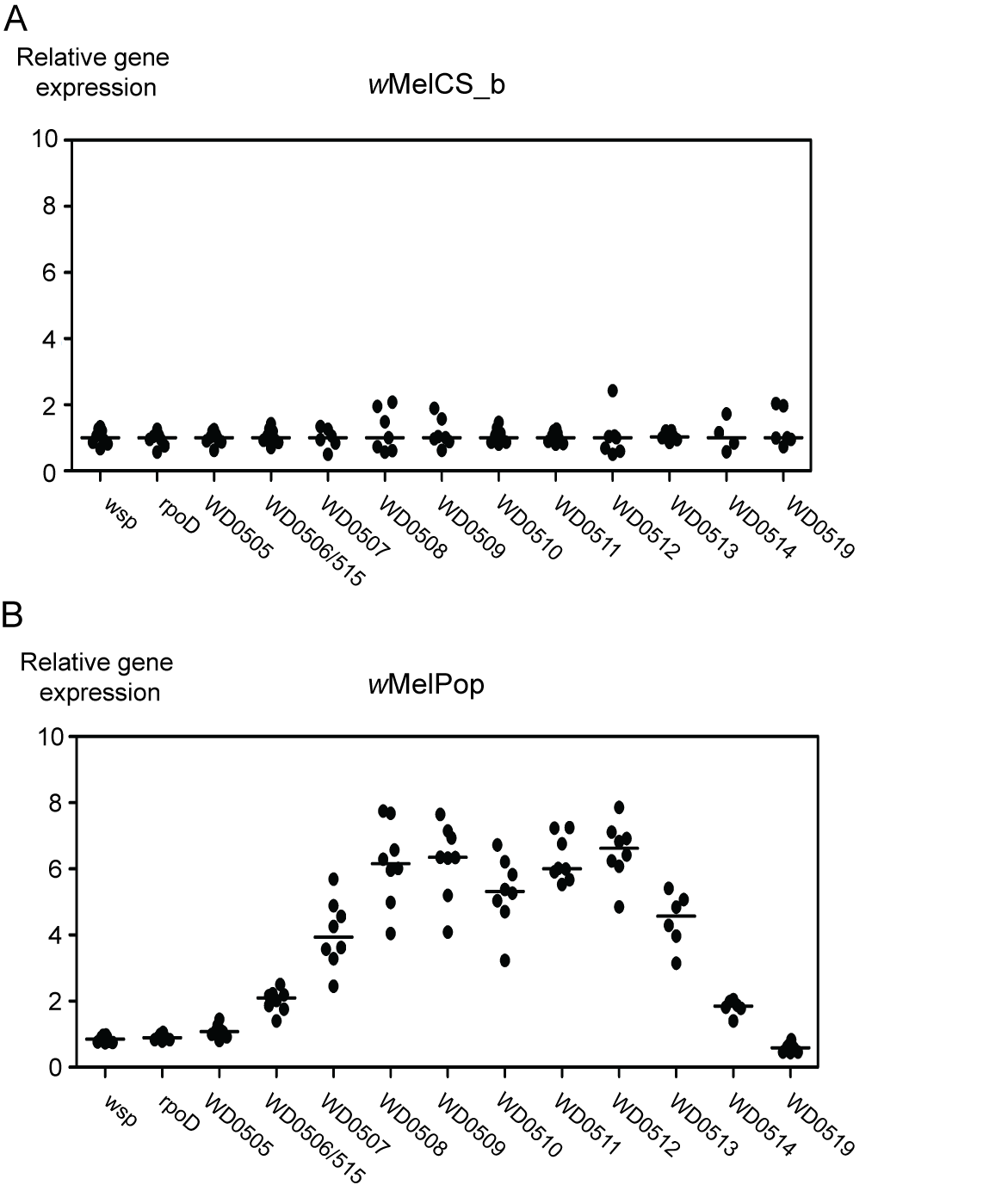

Supplement: S10 Fig — Expression of genes in the Octomom region (WD0507–WD0514), in the flanking repeated region (WD0506/WD0515), in the immediately adjacent region (WD0505 and WD0519), and in other locations of the chromosome (wsp and rpoD) in wMelCS_b (A) and wMelPop (B) (both in DrosDel isogenic background). The expression levels of WD0506–WD0513 are higher in wMelPop than in wMelCS_b (t-test, p < 0.001 for all). The expression levels of Octomom gene WD0514 and genes outside Octomom (wsp, rpoD, WD0505, and WD0519) are not significantly different between the two Wolbachia variants. Relative expression for each gene is calculated using gmk as a reference gene and is relative to that of wMelCS_b samples. RNA was extracted from eight samples of ten 3- to 6-d-old iso males, and real-time qPCR was performed on cDNA with specific primers. Lines are medians of the replicates. Cycle threshold values for the genes WD0507, WD0513, and WD0514 are high, indicating low gene expression levels for these genes. These cycle threshold values fall in a nonlinear section of the standard curve, making the quantification inaccurate. Moreover, cycle threshold values for some reactions were below the detection limit. Supporting data can be found in S27 Data. (TIF) [file pbio.1002065.s037.tif]
