# Supplementary material for: Mutualism Breakdown by Amplification of Wolbachia Genes
Source: PLoS Biol. 2015 Feb 10;13(2):e1002065. doi: 10.1371/journal.pbio.1002065 (PMC4323108; doi:10.1371/journal.pbio.1002065)
Supplement: S1 Table — (DOCX) [file pbio.1002065.s038.docx]

| ***Wolbachia* variant** | **Female genetic background** |
| --- | --- |
| no *Wolbachia* | *iso* |
| *w*MelCS_b | *iso* |
| 1 copy *w*MelPop | *w^1118^* |
| 2 copies *w*MelPop | *w^1118^* |
| ≥10 copies *w*MelPop | *iso* and *w^1118^* |
