# Supplementary material for: Mutualism Breakdown by Amplification of Wolbachia Genes
Source: PLoS Biol. 2015 Feb 10;13(2):e1002065. doi: 10.1371/journal.pbio.1002065 (PMC4323108; doi:10.1371/journal.pbio.1002065)
Supplement: S2 Table — (DOCX) [file pbio.1002065.s039.docx]

| **Figure** | **Generation number** | |
| --- | --- | --- |
|  | ***w^1118^*** | ***iso*** |
| **1B** | 5 | - |
| **3A** | 10 | 14 |
| **3B** | - | 22 |
| **3C** | 11 | 15 |
| **3D** | - | 24 |
| **3E** | 13 | 17 |
| **4** | 44 | 48 |
| **S3** | 44 | - |
| **S5A** | - | 3 |
| **S5B** | - | 5 |
| **S5C** | 3 | - |
| **S5D** | 6 | - |
| **S5E** | 8 | 12 |
| **S5F** | 8 | 12 |
| **S5G** | 10 | 14 |
| **S5H** | 12 | 16 |
| **S6** | - | 22 |
