# Supplementary material for: Mutualism Breakdown by Amplification of Wolbachia Genes
Source: PLoS Biol. 2015 Feb 10;13(2):e1002065. doi: 10.1371/journal.pbio.1002065 (PMC4323108; doi:10.1371/journal.pbio.1002065)
Supplement: S3 Table — (DOCX) [file pbio.1002065.s040.docx]

| **Name** | **Primer sequence (5’- 3’)** |
| --- | --- |
| Link_seq_1 | CCTTCAAGCGAGGAGATTTG |
| Link_seq_2 | GCCTAAAGGAAGCTGCAAAG |
| Link_seq_3 | TTGTTCCCAATCCATACTTTTC |
| Link_seq_4 | TTGGGTCAGCTATTCGTTCC |
| Link_seq_5 | CTACAATATAGTTAATTGG |
| Link_seq_6 | TAACCGCTTCCCCTTGTATG |
| Link_seq_7 | AATGGTTTGTTCCAGGTTGC |
